# Compelling Evidence Linking CD40 Gene With Graves’ Disease in the Chinese Han Population

**DOI:** 10.3389/fendo.2021.759597

**Published:** 2021-11-18

**Authors:** He Jiang, Fei-Fei Yuan, Hai-Ning Wang, Wei Liu, Xiao-Ping Ye, Shao-Ying Yang, Hui-Jun Xie, Sha-Sha Yu, Yu-Ru Ma, Le-Le Zhang, Shuang-Xia Zhao, Huai-Dong Song, The China Consortium for the Genetics of Autoimmune Thyroid Disease

**Affiliations:** ^1^ Liver Cancer Institute, Zhongshan Hospital, Fudan University, Shanghai, China; ^2^ Department of Molecular Diagnostic and Endocrinology, The Core Laboratory in Medical Center of Clinical Research, The Ninth People’s Hospital Affiliated to Shanghai Jiao Tong University School of Medicine, Shanghai, China; ^3^ Department of Endocrinology, The Ninth People’s Hospital Affiliated to Shanghai Jiao Tong University School of Medicine, Shanghai, China

**Keywords:** Graves’ disease, single nucleotide polymorphisms, CD40, association analysis, expression quantitative trait locus

## Abstract

Mutations in *CD40* have been widely reported to be risk factors for Graves’ disease (GD). The gene, along with its cognate ligand CD40L, may regulate pro-inflammatory and immune responses. Rs1883832, located at the -1 position of the Kozak sequence, is the most well-studied single nucleotide polymorphism (SNP) of *CD40*, and has been confirmed to predispose those with the alteration to GD, regardless of ethnicity. Our genome-wide association study (GWAS) indicated that several SNPs, including rs1883832 located within the vicinity of *CD40* were associated with GD in the Han Chinese population. Aiming at identifying the most consequential SNP and its underlying pathogenic mechanism, we performed a two-stage refined study on 8,171 patients with GD and 7,906 controls, and found rs1883832 was the most significantly GD-associated SNP in the *CD40* gene region (*P*_Combined_ = 9.17×10^-11^, OR = 1.18). Through searching the cis-expression quantitative trait locus database and using quantitative RT-PCR, we further discovered that the rs1883832 genotype can influence *CD40* gene transcription. Furthermore, we demonstrated that rs1883832 is a susceptibility locus for pTRAb+ GD patients. In conclusion, the current study provides robust evidence that rs1883832 can regulate CD40 gene expression and affect serum TRAb levels, which ultimately contributes to the development of GD.

## Introduction

Autoimmune thyroid diseases (AITDs) are characterized by autoimmune response and lymphocytic infiltration of the thyroid parenchyma (1, 2). These ailments are the most common autoimmune disorders, and include Graves’ disease (GD) and Hashimoto’s thyroiditis (the prevailing cause of hypothyroidism). GD poses a health risk globally, with a prevalence of ~2% in females and 0.2% in males. People aged 20-50 years are particularly vulnerable (3). While both exogenous factors (e.g., infection, stress, radiation, and iodine intake) and endogenous factors (e.g., biological sex and genetic disposition) both contribute to GD development, twin studies have shown that genetic factors could explain as much as 79% of GD predisposition (4, 5). To date, large-scale genetic studies have identified several susceptibility genes for GD, including thyroid-specific genes (thyroglobulin and thyrotropin receptor) and particularly immune-regulating genes, such as human leukocyte antigen (*HLA*), the protein tyrosine phosphatase nonreceptor type 22 (*PTPN22*), cytotoxic T-lymphocyte associated antigen 4 (*CTLA-4*), cluster of differentiation 40 (*CD40*), secretoglobin family 3A member 2 (*SCGB3A2*), Fc receptor-like 3 (*FCRL3*), BTB domain and CNC homolog 2 (*BACH2*), and integral membrane protein 2A (*ITM2A*) (6–17).

The *CD40* gene is a member of the tumor necrosis factor (TNF) receptor superfamily. The encoded protein is mainly expressed on B cells and different antigen-presenting cells (APCs), which is essential for mediating immune and inflammatory responses, including T cell-dependent immunoglobulin class switching, B cell proliferation and activation, and memory B cell development (2, 18). It has been reported that CD40 is also expressed on some nonimmune cells, such as pancreatic beta cells, endothelial cells, and thyroid epithelial cells (19–22). CD40 interacts with its ligand, CD40L (CD154), and plays a prominent role in many autoimmune diseases, such as rheumatoid arthritis (RA), multiple sclerosis (MS), and systemic lupus erythematosus (SLE) (23–25). Most investigators agree that GD is an organ-specific autoimmune disorder mediated by B and T cells, due to a complex interplay of factors that lead to the loss of immune tolerance to thyroid antigens and to the initiation of a sustained autoimmune reaction (2, 26). Given that *CD40* plays an important role in humoral and cellular immunity, it is easy to understand why it is likely that *CD40* contributes to GD development.

Linkage analyses have confirmed the genetic association of *CD40* with GD. Rs1883832, the most well-studied SNP located at position -1 in the Kozak sequence of the *CD40* gene, was reported to be associated with GD with a relative risk ranging from 1.22-1.93 in different ethnicities (12, 27–29). Moreover, investigators have verified that the C allele of rs1883832 could promote the translation of *CD40* and lead to GD (30). As for Chinese Han population, Wang et al. genotyped rs1883832 in 196 GD cases and 122 controls and concluded there is an association between rs1883832 and GD susceptibility (OR = 1.57) (31). However, most researches have used candidate gene strategies or have performed replication studies to verify the relationship between *CD40* and GD. The generalizability of the Wang et al. findings is constrained by the relatively small sample size and the enlistment of a single patient center. Therefore, their results need to be confirmed in large-scale and multi-center genetic study, potentially using a different strategy.

Genetic underpinnings are of fundamental importance for determining individual differences in immune and inflammatory responses. Fairfax et al. (32) assessed the correlation between SNP genotypes and gene transcription levels in monocytes under lipopolysaccharide (LPS) or interferon-γ (IFN-γ) stimulation compared with those in the naïve state. They established a database containing SNPs and the correlated genes within a 1 Mb window ─ the cis-expression quantitative trait locus (cis-eQTL) database (32). Molecular, cellular, and environmental factors all contribute to the pathogenesis of autoimmune diseases. This database makes it possible to explore functional genetic variants and their modulated genes.

Our group conducted a genome-wide association study (GWAS) and imputation analysis in 1,536 GD patients and 1,516 controls, and confirmed that *HLA*, *CTLA4*, *FCRL3*, and *THSR* were susceptibility genes for GD and identified two new GD risk loci in *RNASET2* and *GDCG4P14* in Chinese Han population (8). Rs1883832 showed a weak association with GD in our GWAS data. In order to identify the causal loci in the *CD40* gene region and the underlying pathogenic mechanism, we performed a two-stage genetic analysis in a large cohort (n =16,077) and provided compelling evidence for the association of rs1883832 with GD. We also confirmed the relationship between rs1883832 genotypes and *CD40* transcriptional levels by searching through the aforementioned cis-eQTL database and the Genotype-Tissue Expression (GTEx) database, and also by applying qRT-PCR method.

## Materials and Methods

### Subjects

All individuals were Chinese Han population and were recruited by The China Consortium for the Genetics of Autoimmune Thyroid Disease. This study was performed in accordance with the principles of the Declaration of Helsinki. Approval was granted by the local ethics committees of all partner hospitals and all participants provided written informed consent prior to participation. In the initial GWAS stage, 1,536 GD and 1,516 sex-matched controls were included and 1,442 GD cases and 1,468 controls remained after quality control (8). In the replication stage, we recruited a further 6,729 cases and 6,438 controls. The inclusion and exclusion criteria were as previously described (8, 15). The plasma level of thyroid stimulating hormone receptor autoantibody (TRAb) in GD patients treated with antithyroid drugs (ATD) for ≥ 1 year were analyzed by an enzyme-linked immunosorbent assay ELISA kit, and we defined TRAb ≥ 1.5 U/L as persistent TRAb positive (pTRAb+) and TRAb < 1.5 U/L as non-persistent TRAb-positive (pTRAb−) (8, 16). Sample characteristics are presented in (**Table 1**).

**Table 1:** Sample characteristics of the current study.

Genotyping stage	Disease status	No.	Sex (M/F)	Age
GWAS stage	GD (GO)	1,442 (609)	335/1,107	39 ± 14
	Control	1,468	359/1,109	45 ± 9
Replication stage	GD (GO)	6,729 (1,079)	1,562/5,167	39 ± 14
	Control	6,438	1,732/4,706	47 ± 12
Combined stage	GD (GO)	8,171 (1,688)	1,897/6,274	39 ± 14
	Control	7,906	2,091/5,815	45 ± 9

GO, Graves ophthalmopathy; F, female; M, male.

### SNP Selection for the Replication Stage and Sample Size Calculation

Based upon our GWAS data and the HapMap recombination rates in 20q13.12, a 400-kb chromosomal region was chosen for the current study. We used Haploview (version 4.2) to select eight candidate tag SNPs from 49 SNPs with P_GWAS_< 0.05 according to their linkage disequilibrium information. Logistic analysis suggested that among the tag SNPs, only rs1883832 needed to be genotyped in a replication cohort. To confirm the genome-wide significant difference of rs1883832 at above 80% power, we calculated the necessary sample size using QUANTO (version 1.2.4) and found a minimum of 1,849 case-control pairs would be needed.

### Genotyping

The GWAS study was performed using Illumina660-Quad Bead-Chips (Illumina, San Diego, CA, USA) at the Chinese National Human Genome Center in Shanghai, China [8]. Genotype cluster analysis was conducted using Illumina BeadStudio 3.3 software (Illumina, San Diego, CA, USA). In the replication stage, rs1883832 was genotyped using TaqMan SNP Genotyping Assays on an ABI ViiA 7 Real-Time PCR instrument (Applied Biosystems, Foster City, CA, USA).

### The Correlation Analysis of eSNPs for *CD40* and GD

Fairfax et al. (32) investigated the relationship between gene transcription levels and 609,704 SNPs through exposing the CD14+ monocytes of 367 individuals to interferon-γ (IFN-γ) (20 ng/ml, 24 h), 322 individuals to lipopolysaccharide (20 ng/ml, 24 h), 261 individuals to LPS (20 ng/ml, 2 h), and 414 individuals to naïve state. Data across the above four conditions were available from 228 individuals, thus permitting cross-treatment comparison (32). The eQTL analysis was also conducted using the Genotype-Tissue Expression (GTEx) portal (https://gtexportal.org/home/). The GTEx project has generated rich transcriptome data in a variety of human tissue types, thus providing insights into the regulatory role of genetic variation (33). Through searching these two databases, we investigated the expression SNPs (eSNPs) for *CD40* and analyzed whether these eSNPs were associated with Graves’ disease based on the GWAS data.

### Sorting of Leukocytes and Real-Time RT-PCR

Ninety-five healthy controls were genotyped using Taqman assays (Applied Biosystems, Foster City, CA, USA) on a ViiA 7 Real-Time PCR System (Thermo Fisher Scientific, Waltham, MA, USA) and their peripheral blood mononuclear cells (PBMCs) were isolated using the density gradient separation method. The leukocyte subtypes of CD4+ T-cells, CD8+ T-cells, CD19+ B-cells and CD14+ monocytes were isolated using MACS cell separation kits (CD4+ T Cell Isolation Kit, CD8+ T Cell Isolation Kit, CD19 Microbeads and CD14 Microbeads) (Miltenyi Biotec, Bergisch Gladbach, Germany) and an autoMACS Separator (Miltenyi Biotec, Bergisch Gladbach, Germany). Only PBMCs and subpopulations with a purity of greater than 90% were selected for the follow-up expression experiments (8). Expression of *CD40* was determined by quantitative real-time PCR, and GAPDH was used as an internal reference.

### Statistical Analysis

The association analysis for GWAS and the replication stage were performed using the PLINK Cochran-Armitage trend test and logistic regression (34). For the combined stage and TRAb +/- subsets analysis, we used the Cochran–Mantel–Haenszel stratification test (34). The forward and two-locus logistic regression analyses were conducted using the R software environment and PLINK. The regional plots were generated by LocusZoom (http://locuszoom.sph.umich.edu/). The CD40 expression data were analyzed using ANOVA and the unpaired Student’s t-test.

## Results

### Refining Association Study in the 20q13.12 Region

**Figure 1:**
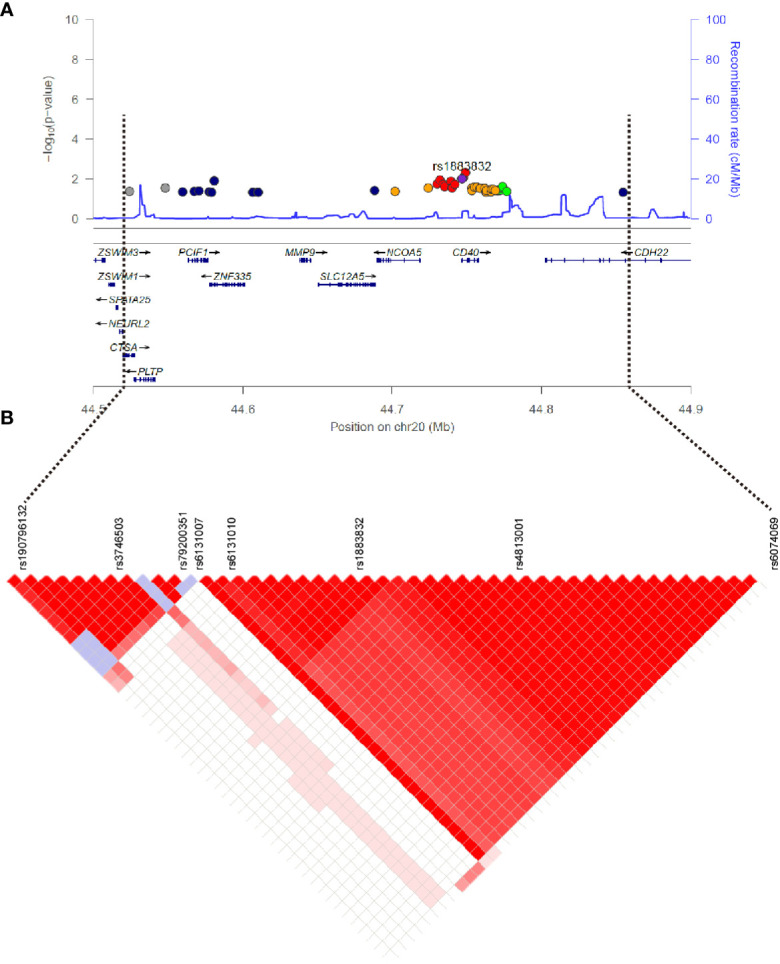
Regional plots of association results on chr20q.13.12 and linkage disequilibrium analysis. **(A)** The 49 SNPs associated with GD in the GWAS stage. The color of each dot reflects its r^2^ value with the rs1883832, which is represented by the purple dot. **(B)** Linkage disequilibrium analysis for the 49 SNPs.

**Figure 2:**
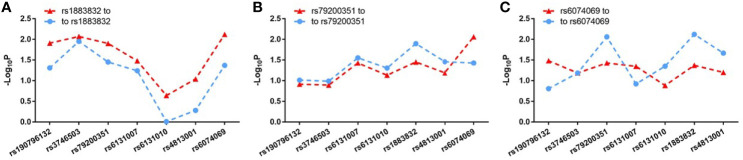
Two-locus logistic regression results for three tag SNPs in the GWAS stage. In the GWAS stage, P values of the other seven tag SNPs after conditioning on rs1883832 **(A)**, rs79200351 **(B)** or rs6074069 **(C)** are indicated by blue dots and P values of rs1883832 **(A)**, rs79200351 **(B)** or rs6074069 **(C)** after condition on the other seven tag SNPs are indicated in red triangles.

Evidence for the strong linkage of *CD40* to GD had been reported through whole genome linkage scanning, and rs1883832 appeared to be the causal SNP of GD in the vicinity of *CD40* (12, 27–29, 31). To deeply analyze the relationship between *CD40* and GD, we performed a genome-wide association study (GWAS) and imputation analysis of 1,442 GD cases and 1,468 controls after quality control (**Table 1**). Because SNPs surrounding the *CD40* gene exhibited significant association with GD, we selected a 400 kb region on 20q13.12, which includes *CD40*, and conducted a refining study aimed at providing more compelling evidence for GD-associated SNPs. After filtering SNPs with minor allele frequency (MAF) < 0.01, 761 SNPs remained, and of them, 49 SNPs were associated with GD (*P*_GWAS_ < 0.05) (**Figure 1A** and **Table S1**). Through analyzing the linkage disequilibrium (LD) structure of the 49 GD-associated SNPs, we selected eight tag SNPs to further narrow the linked chromosomal regions (**Figure 1B** and **Table S2**). Of these, forward logistic regression analysis showed that rs79200351, rs6074069, and rs1883832 yielded evidence of association (*P*_forward_ = 0.004, 0.004, 0.008, respectively). These three SNPs are located in three different LD blocks (**Figure 1B**), and of them, rs1883832 displayed the strongest association with GD (P = 9.77×10^-3^). Unlike the other two candidates, two-locus logistic regression indicated that rs1883832 improved the regression models of the other seven tag SNPs, while acting as an independent GD-associated SNP (**Figure 2**). Therefore, only rs1883832 was tested for replication in an additional 6,729 GD cases and 6,438 controls (**Table 2**) and showed a consistent association with GD in both stages (*P*_Replication_ = 4.95×10^-10^, OR = 1.18; *P*_Combined_ = 9.17×10^-11^, OR = 1.18). Taken together, we concluded that rs1883832 is the most strongly GD-associated SNP in the 20q13.12 region.

**Table 2:** The association analysis results of rs1883832 in three stages.

CHR	SNP	Position	Annotated Gene	Risk Allele	GWAS Stage (1,442 *vs*. 1,468)				Replication Stage (6,729 *vs*. 6,438)				Combined Stage (8,171 *vs*. 7,906)			
					Case RAF	Control RAF	*P* value	OR (95%CI)	Case RAF	Control RAF	*P* value	OR (95%CI)	Case RAF	Control RAF	*P* value	OR (95%CI)
20	rs1883832	44746982	*CD40*	C	0.67	0.64	9.77E-03	1.15 (1.03-1.28)	0.68	0.64	4.95E-10	1.18 (1.12-1.24)	0.68	0.64	9.17E-11	1.18 (1.12-1.24)

RAF, risk allele frequency; F, female; M, male.

### The Effects of rs1883832 on *CD40* Expression

**Figure 3:**
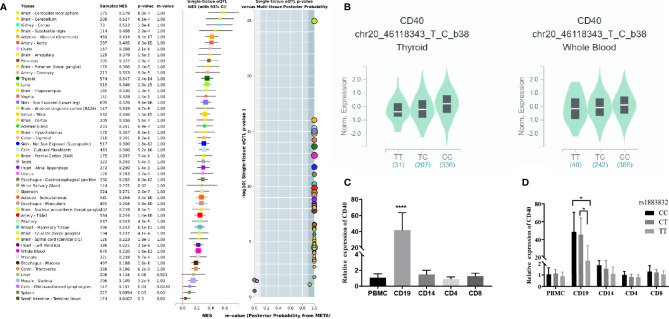
The cis-eQTL analysis of rs1883832 and the expression analysis of *CD40* gene in PBMCs and cell subsets. **(A)** Analysis of the relationship between rs1883832 and *CD40* mRNA levels in different tissues. **(B)** The relationship between the C allele of rs1883832 and *CD40* mRNA levels in thyroid and the whole blood samples. **(C)** Relative mRNA expression levels of *CD40* in PBMCs and the four cell subtypes. **(D)** Relative mRNA expression levels of *CD40* for different genotypes of rs1883832 in PBMC and the four cell subtypes. Results are arranged by the subset of PBMCs from 95 individuals. *P < 0.05; ****P < 0.001.

Jacobson et al. reported that the C allele of rs1883832 predisposes one to develop GD by increasing the translation efficiency of *CD40* mRNA, rather than operating at the transcription level (30). Unfortunately, their study was based on a small sample size, from which one cannot draw a comprehensive conclusion. We therefore speculated that the true relationship between rs1883832 and *CD40* mRNA expression level still needs to be determined. First, by searching cis-eQTL data from the GTEx database, we found that rs1883832 is correlated with *CD40* expression within a cluster of tissues, including the thyroid and whole blood (**Figure 3A**). Risk allele C of rs1883832 significantly upregulated *CD40* mRNA levels both in 670 whole blood and 574 thyroid samples from the GTEx database, as assessed by cis-eQTL analysis (*P* = 1.6×10^-13^ and 2.4×10^-14^, Normalized effect size (NES) = 0.220 and 0.347, respectively) (**Figures 3A, B**). Furthermore, we searched the cis-eQTL database built by Fairfax et al. (32) and found 77 SNPs were correlated with *CD40* expression. After quality control, 40 SNPs were included in our own GWAS data, and eight SNPs among them were associated with GD (P < 0.05, **Table 3**). SNPs that influence gene expression were called eSNPs. Interestingly, rs1569723, rs1883832, rs4810485, and rs6074022 exhibited stronger association with *CD40* expression and GD than rs4810486, rs4813003, rs1883835, and rs2143699. Compared with the naïve state, rs1569723, rs1883832, rs4810485, and rs6074022 showed weaker correlation with *CD40* expression when stimulated by IFN-γ or LPS. After analyzing the LD structure of the eight eSNPs, we found that rs1883832 could capture rs1569723, rs4810485, and rs6074022 with r^2^ > 0.8, and rs4813001 (one of the eight tag SNPs mentioned earlier) could capture rs4810486, rs4813003, rs1883835, and rs2143699 with r^2^ > 0.8. However, rs4813001 was filtered out by forward logistic regression in the GWAS stage, and rs1883832 could significantly improve the regression model with rs4813001 (**Figure 2**).

**Table 3:** Stimulated-eQTL results of *CD40*.

SNP	Position	Gene	*P*_GWAS_	*P*_LPS2_	*P*_LPS24_	*P*_IFN-γ24_	*P*_Naive_	Min.dataset
rs1569723	44742064	CD40	1.92E-02	6.97E-09	2.25E-17	2.05E-10	7.86E-22	Naive
rs1883832	44746982	CD40	9.77E-03	6.97E-09	2.25E-17	2.05E-10	7.86E-22	Naive
rs4810485	44747947	CD40	8.69E-03	6.97E-09	2.25E-17	2.05E-10	7.86E-22	Naive
rs6074022	44740196	CD40	2.83E-02	6.97E-09	2.25E-17	2.05E-10	7.86E-22	Naive
rs4810486	44766403	CD40	4.67E-02	2.21E-06	7.45E-12	5.56E-10	8.09E-10	LPS24
rs4813003	44763284	CD40	3.94E-02	2.21E-06	7.45E-12	5.56E-10	8.09E-10	LPS24
rs1883835	44773987	CD40	2.90E-02	1.51E-04	2.78E-11	1.87E-08	1.28E-08	LPS24
rs2143699	44759166	CD40	3.05E-02	1.51E-04	2.78E-11	1.87E-08	1.28E-08	LPS24

Naive, monocytes without LPS or IFN treatment; P_LPS2_, P value of each SNP-regulated CD40 gene expression in monocytes treated with LPS for 2 hours; P_LPS24_, P value of monocytes treated with LPS for 24 hours; P_IFN-γ24_, P value of monocytes treated with IFN-γ for 24 hours.

Finally, we evaluated the *CD40* mRNA level and its correlation with rs1883832 genotypes in different subtypes of peripheral blood mononuclear cell (PBMC), including CD4+ T cells, CD8+ T cells, CD14+ monocytes, and CD19+ B-cells. The results showed that all PBMC subtypes expressed *CD40* while CD19+ B-cells exhibited the highest expression level (**Figure 3C**), and CC/CT genotypes of rs1883832 had higher *CD40* expression than the TT genotype in CD19+ B-cells (P <0.05 in **Figure 3D**). Thus, we speculated that rs1883832 and its linked SNPs probably contribute to the susceptibility for GD by regulating *CD40* expression.

### Association Analysis of rs1883832 in pTRAb +/- GD Subsets

The serum level of thyroid stimulating hormone receptor autoantibodies (TRAb) is a diagnostic and prognostic factor in GD, which can help in monitoring the efficiency of antithyroid drug (ATD) therapy for GD. In this study, we performed an association analysis of rs1883832 in TRAb+/- GD subsets in order to investigate whether rs1883832 could affect the serum level of TRAb, thereby possibly affecting the maintenance of hyperthyroidism. GD patients with TRAb > 1.5 U/L were defined as the persistent TRAb+ group (n = 2,389) and TRAb < 1.5 U/L were as persistent TRAb- group (n = 1,004). Our results showed that rs1883832 was associated with pTRAb+ patients (P = 3.75 × 10^−5^), but was not associated with pTRAb- patients (P = 0.06) (**Table 4**). Furthermore, we performed heterogeneity analysis between the 2,389 pTRAb+ patients and 1,004 pTRAb- patients and found rs1883832 exhibits non-significant heterogeneity (P_h_ = 0.35) between the two subgroups.

**Table 4:** Association analysis of rs1883832 with TRAb subsets in GD patients.

CHR	SNP	Position	Annotated Gene	Risk allele	pTRAb+ (2,389 cases *vs*. 7,866 controls)				pTRAb- (1,004 cases *vs*. 7,866 controls)				*P*_h_
					Case RAF	Control RAF	*P* value	OR (95% CI)	Case RAF	Control RAF	*P* value	OR (95% CI)	
20	rs1883832	44746982	*CD40*	C	0.67	0.64	3.75E-05	1.16 (1.08-1.25)	0.66	0.64	6.00E-02	1.10 (1.00-1.22)	3.50E-01

RAF, risk allele frequency; P_h_, the allele frequency heterogeneity of rs1883832 in pTRAb+ and pTRAb− patients.

## Discussion

By using our GWAS data and the tag SNP selection strategy, we conducted a refined association study of the region surrounding the *CD40* gene and found rs1883832 was the most relevant SNP statistically associated with GD. Based on the GTEx database and the cis-eQTL database built by Fairfax et al. (32), we analyzed the SNPs relevant to *CD40* expression and found rs1883832 as well as its tightly linked SNPs exhibited a strong association with *CD40* expression. In addition, as a functional polymorphism located at the -1 position in the Kozak sequence of the *CD40* gene, rs1883832 was demonstrated to be associated with *CD40* mRNA expression level in PBMCs in our study. These results indicated that rs1883832 probably regulated *CD40* expression, thereby contributing to the pathogenesis of GD. Furthermore, the allele frequency of rs1883832 in pTRAb+/pTRAb- patients had no significant difference (P_h_ = 0.35), but the association analysis showed that rs1883832 was correlated with pTRAb+ patients only (P = 3.75 × 10^−5^). Therefore, we concluded that rs1883832 participated in the development of GD.

CD40 is a type I integral membrane glycoprotein and a cell-surface member of TNF receptor superfamily that functions in B-cell proliferation and activation, T cell priming, antigen presentation, immunoglobulin isotype switching, germinal centers development and humoral immune memory (18, 35). CD40 interacting with its ligand CD154 (CD40L) could trigger immune and inflammatory responses, and it has been found to be associated with several autoimmune diseases such as GD and Hashimoto’s thyroiditis (2), systemic lupus erythematosus (SLE) (36), rheumatoid arthritis (RA) (37) and multiple sclerosis (38). The linkage and association analysis has provided robust evidence that CD40 could confer susceptibility to GD. Tomer firstly identified rs1883832 (CC genotype) and CD40 were both associated with GD in Caucasian population (12). After that, rs1883832 CC genotype was proved to be a susceptibility variant in Japanese GD patients (12, 28), and the C allele was demonstrated to raise the risk of GD in the Chinese Han population (39, 40). However, these studies used candidate gene strategies or performed replication studies in relatively small sample sizes, which have limited statistical power. In contrast, we provide more reliable and unbiased results that rs1883832 is probably the causal SNP located in the *CD40* gene region through a GWAS strategy, and two-stage refined association analysis in large Chinese cohorts.

Given that rs1883832 is located at the -1 position of the Kozak consensus sequence of *CD40*, variations in this sequence are supposed to affect gene translation (41). Jacobson et al. used a battery of methods including *in vitro* transcription-translation assays, surface expression analysis of cells transfected with the two alleles, and analyses of B cells from individuals with different SNP genotypes, to demonstrated that the C allele of rs1883832 was correlated with increased *CD40* translational efficiency, compared with the T allele (30). However, they also concluded that there was no correlation between rs1883832 genotype and *CD40* mRNA levels by using quantitative RT-PCR data from the purified B cells (cultured for 16h with or without interferon-γ) of 11 individuals (30). These results might require further confirmation by increasing the number of patients and/or using other methods. Recently, *CD40* expression has been detected on thyroid epithelial cells (19, 42), and several studies unraveled the mechanism how *CD40* contributes to GD pathogenesis. Jacobson et al. revealed a stronger association of rs1883832 genotype CC with persistently elevated thyroid antibody among GD patients than those who were thyroid antibody negative, with significant expression of *CD40* mRNA and corresponding proteins in the thyroid observed(GD target tissue) (43). They proposed *CD40* overexpression on thyrocytes augmented thyroid-directed autoimmunity through two possible mechanisms. The extrinsic mechanism is based on the fact that thyrocytes can present self-peptides within HLA class II molecules to intra-thyroidal T cells under certain conditions, which makes surface molecules like CD40 transmit co-stimulatory signals, finally leads to T cells activation. The intrinsic mechanism, through the activation of *CD40* signaling pathway within the thyrocytes could alter their physiology, lead to inflammation and autoimmunity (43). Using the transgenic mouse model overexpressing *CD40* in the thyroid, Huber et al. demonstrated that thyroidal *CD40* overexpression augmented the production of thyroid-specific Abs due to the activation of downstream cytokines and chemokines (most notably IL-6), resulting in more severe experimental autoimmune GD (44). Recently, a pilot study on a small cohort of 13 GD patients demonstrated that specific *CD40* haplotypes composed of six SNPs were associated with higher *CD40* mRNA levels and clinical response to Iscalimab (the anti-CD40 monoclonal antibody). As one of the key SNPs, rs1883832 could differentiate responders from non-responders – C allele associated with response to Iscalimab and T allele with no response (45). Therefore, we evaluated the relationship between rs1883832 genotype and *CD40* mRNA levels in PBMC based a study of 95 healthy people, and confirmed that individuals with CC and CT genotypes had higher *CD40* mRNA levels in CD19+ B-cells. Our results provide more insights into how the genetic variations at the *CD40* gene-locus could affect the clinical response of *CD40*-targeted therapies.

Polymorphisms located within immune regulator gene regions are associated with a variety of diseases (30). Through exposing primary monocytes from 432 healthy volunteers to IFN-γ or LPS and mapping gene expression as a quantitative trait loci, Fairfax et al. established a cis-eQTL database that can help to understand the nature and functional consequences of genetic variation (32). Using this database, we found eight GD-associated SNPs were correlated with CD40 expression in the naïve or stimulus state (**Table 3**). However, rs1883832 with its highly linked SNPs (r^2^ > 0.8) exhibited a stronger association than the other seven SNPs under all conditions

TRAb is implicated in GD pathogenesis, and its presence in serum is diagnostic for Graves’ disease (46). TRAb is also related with the extrathyroidal manifestations of GD (such as Graves’ ophthalmopathy and pretibial dermopathy) (47, 48). Our group has performed association analysis for several SNPs in pTRAb+/- subgroups (8, 16), but so far, no studies have reported the relationship between rs1883832 and TRAb. Therefore, our results provide a new view of the involvement of rs1883832 in GD pathogenesis.

In summary, by means of a refined study including 8,171 GD patients and 7,906 controls, our research provides compelling evidence that rs1883832 is the most significant GD-associated SNP located within the CD40 gene region in the Chinese Han population, and is also a susceptibility locus for pTRAb+ GD patients. Furthermore, monocytes and CD19+ B-cells carrying different rs1883832 genotypes showed distinct *CD40* mRNA levels, which indicates that rs1883832 and its highly linked SNPs probably affect the CD40 gene at the transcriptional level. Considering that rs1883832 genotypes influence the translation of *CD40*, we propose that through transcriptional and translational pathophysiological aspects, rs1883832 alters *CD40* gene expression, which ultimately contributes to Graves’ disease etiology.

## Data Availability Statement

The original contributions presented in the study are publicly available. This data can be found here: https://wwwdev.ebi.ac.uk/eva/?eva-study=PRJEB48200.

## Ethics Statement

The studies involving human participants were reviewed and approved by the ethics committees of all partner hospitals including the Shanghai ninth peoples’ hospital, the first affiliated hospital of Bengbu medical college, the Linyi hospital and the Xuzhou central hospital. The patients/participants provided their written informed consent to participate in this study.

## Author Contributions

H-DS and S-XZ conceived and designed the project. HJ, F-FY, and WL contributed to the project management and the replication genotyping. HJ and F-FY took part in the statistical analysis. HJ, F-FY, WL, H-NW, X-PY, S-YY, H-JX, S-SY, Y-RM, and L-LZ took part in the clinical samples collection, DNA extraction, and sample quality control HJ, F-FY, and WL wrote the manuscript. All authors contributed to the article and approved the submitted version.

## Funding

This study was supported by grants from the National Natural Science Foundation of China (No. 81870537, No. 81770786, No. 81200643 and No. 31501015) and the Shanghai Municipal Education Commission-Gaofeng Clinical Medicine (No. 20161318).

## Conflict of Interest

The authors declare that the research was conducted in the absence of any commercial or financial relationships that could be construed as a potential conflict of interest.

## Publisher’s Note

All claims expressed in this article are solely those of the authors and do not necessarily represent those of their affiliated organizations, or those of the publisher, the editors and the reviewers. Any product that may be evaluated in this article, or claim that may be made by its manufacturer, is not guaranteed or endorsed by the publisher.
