# Supplementary figures and images for: Compelling Evidence Linking CD40 Gene With Graves’ Disease in the Chinese Han Population
Source: Front Endocrinol (Lausanne). 2021 Nov 18;12:759597. doi: 10.3389/fendo.2021.759597 (PMC8639283; doi:10.3389/fendo.2021.759597)

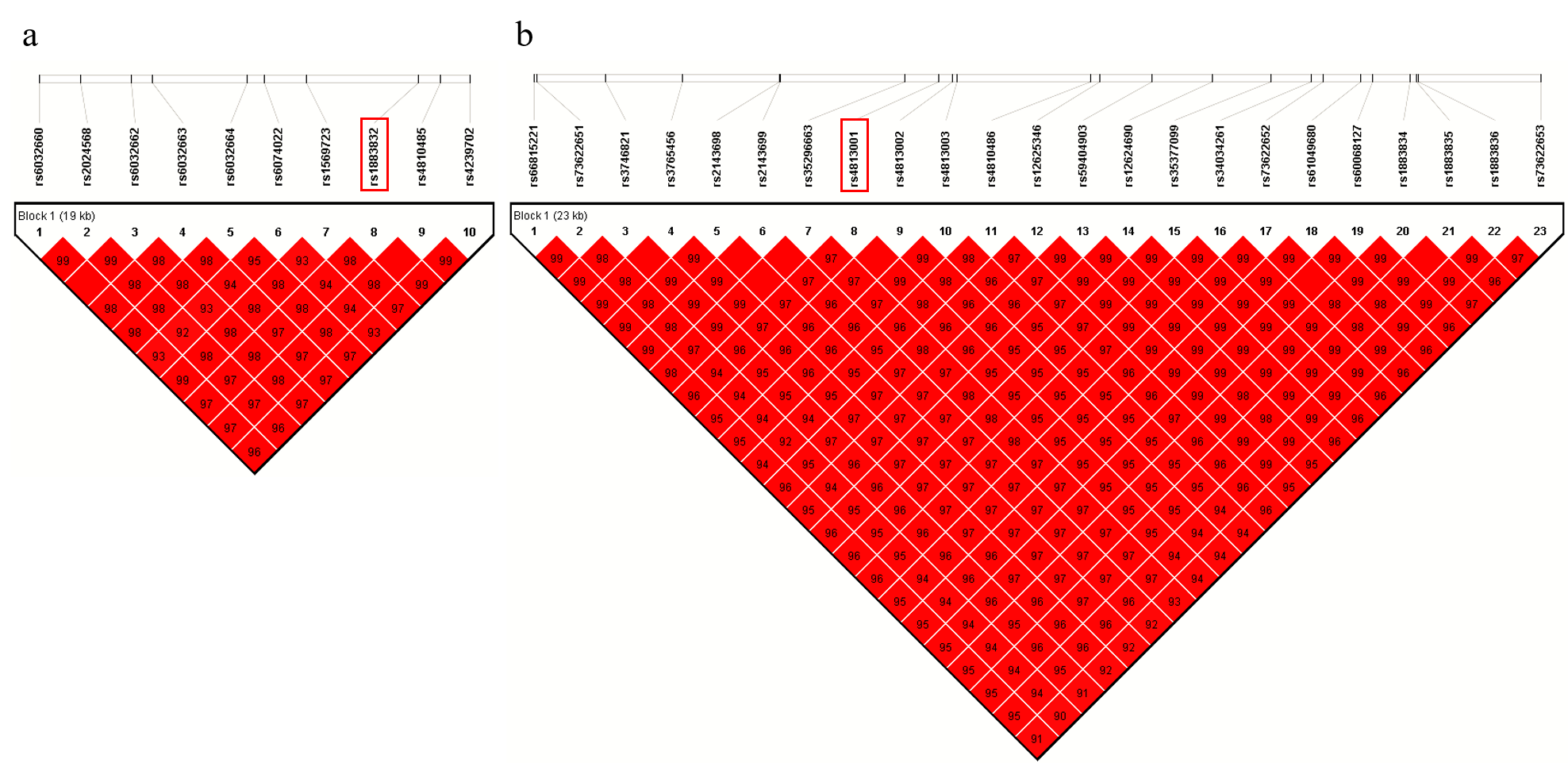

Supplement: Supplementary file 1 [file Image_1.jpeg]
